# Carbon Nanostructures, Nanolayers, and Their Composites

**DOI:** 10.3390/nano11092368

**Published:** 2021-09-12

**Authors:** Nikola Slepičková Kasálková, Petr Slepička, Václav Švorčík

**Affiliations:** Department of Solid State Engineering, University of Chemistry and Technology Prague, 166 28 Prague, Czech Republic; nikola.kasalkova@vscht.cz (N.S.K.); vaclav.svorcik@vscht.cz (V.Š.)

**Keywords:** carbon, nanoparticles, nanostructures, thin layers, composites, Q-carbon, graphene, DLC layers, modification, application

## Abstract

The versatility of the arrangement of C atoms with the formation of different allotropes and phases has led to the discovery of several new structures with unique properties. Carbon nanomaterials are currently very attractive nanomaterials due to their unique physical, chemical, and biological properties. One of these is the development of superconductivity, for example, in graphite intercalated superconductors, single-walled carbon nanotubes, B-doped diamond, etc. Not only various forms of carbon materials but also carbon-related materials have aroused extraordinary theoretical and experimental interest. Hybrid carbon materials are good candidates for high current densities at low applied electric fields due to their negative electron affinity. The right combination of two different nanostructures, CNF or carbon nanotubes and nanoparticles, has led to some very interesting sensors with applications in electrochemical biosensors, biomolecules, and pharmaceutical compounds. Carbon materials have a number of unique properties. In order to increase their potential application and applicability in different industries and under different conditions, they are often combined with other types of material (most often polymers or metals). The resulting composite materials have significantly improved properties.

## 1. Introduction

Carbon is a unique element that can form various compounds and structures on both a macroscopic and nanoscopic scale. More than 95% of known chemical compounds can be classified as carbon-based compounds. This is due to four valence electrons (2s and 2p), which participate in the formation of a bond (single, double, and triple). In addition, carbon can react to form stable substances with more electronegative and electropositive elements. The resulting diversity of the final compounds and nanostructures is accompanied by a vast range of different chemical, physical, and biological properties. This makes carbon one of the most extended and most researched elements in materials science and research [1,2,3,4,5,6].

**Figure 1:**
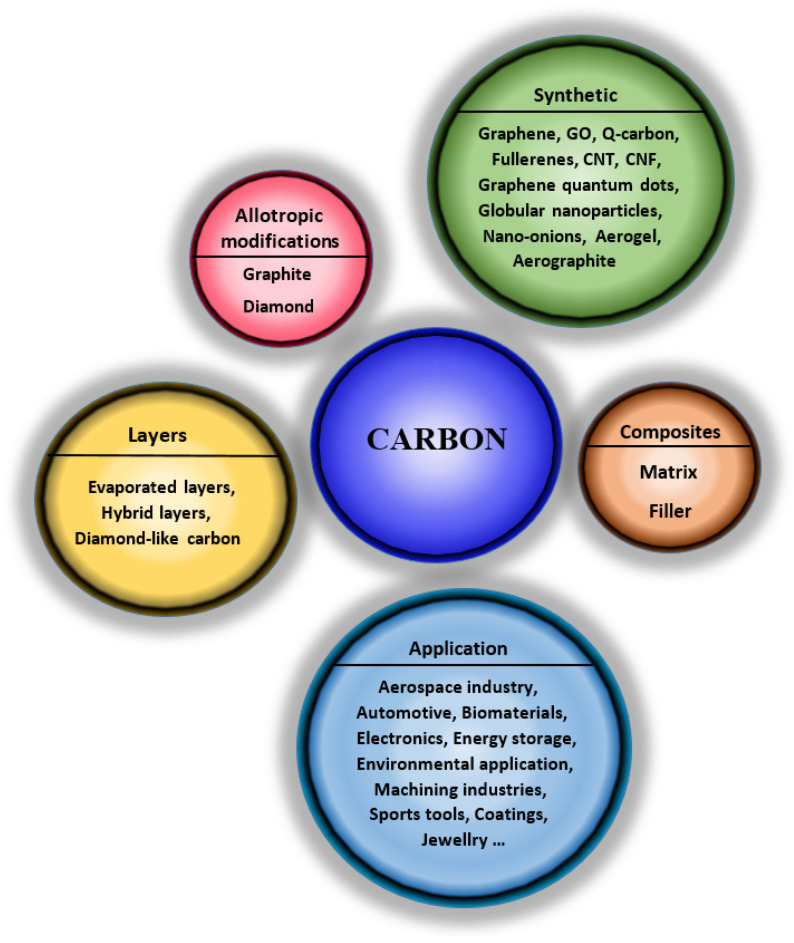
Graphical schema of review idea—carbon nanostructures, carbon-based materials, carbon composites, and their properties and application.

Carbon-based materials play an essential role in many scientific and industrial sectors. Carbon can exist in three different hybridization forms (sp, sp2, and sp3), which allows the creation of many different amorphous and crystalline structures and various forms and dimensions, such as 0D fullerene molecules [7], also usable in different structures: 1D—single and multi-walled carbon nanotubes (CNT) and nanofibers (CNF); 2D—carbon nanohorns [8] and graphene [9]; 3D—foams, aerogel, and hydrogel. Furthermore, the versatility of the arrangement of C atoms to form different allotropes and phases makes carbon a material used in almost all industries (Figure 1) [4]. Although graphite and diamond are allotropes that have been studied and described in great detail, they cannot be omitted in this text on specific carbon structures. This review brings a comprehensive summary of specific carbon nanostructures, their composites, and their applications, which is valuable information for all those involved in both material engineering and tissue engineering fields of research.

## 2. Carbon Nanostructures and Their Composites

### 2.1. Graphite

Graphite is a natural material occurring in crystalline, amorphous, lumpy, or veined forms. It is the most stable form of carbon on Earth and, ideally, consists of endless layers of sp2 hybridized carbon atoms. The individual layers are called “graphene sheets” or “graphene layers”, which are bound together only by weak Van der Waals forces, which give graphite its softness, flexibility, and fissility [1,10,11]. Therefore, it is used as a lubricant in many industries. Thanks to the delocalized cloud of π-electrons it is also an excellent thermal and electrical conductor, but only in the direction of the planes; in the direction perpendicular to the planes, the conductivity is lower. Graphite reflects visible light very well. This can be used to approximate an ideal black body—carbon blacks can absorb up to 99.5% of radiation. Polycrystalline forms of carbon and their black color and minimal particle size allow repeated reflections of light radiation inside the pores and in the gaps between the particles, which leads to gradual absorption. The use of graphite is extensive; graphite rods are used in nuclear reactors [10], and graphite is used as electrodes in dry batteries, serves as reinforcement in steels, and has applications in anti-corrosion layers, etc.

### 2.2. Diamond

A diamond is an allotrope formed by a network of sp3 hybridized carbon atoms arranged in a cubic area-centered lattice. Each carbon atom is bonded to the surrounding four atoms in the form of a tetrahedron. The diamond exists in two modifications—the hexagonal type and the cubic type—while the second type is more abundant. Compared to graphite, diamond is less stable at room temperature and atmospheric pressure, so its synthesis from graphite is very demanding and requires extreme conditions. The reason for the complexity of graphite diamond production is the high activation barrier that separates the two phases, thus preventing their conversion at room temperature and pressure. However, this high energy barrier is behind the existence of diamond; once it is created, the energy barrier prevents it from spontaneously transforming into more thermodynamically stable graphite. Therefore, diamond is called metastable, i.e., kinetically stable, but thermodynamically unstable [12]. It is the hardest known natural substance. Its excellent optical properties make it one of the most sought-after gems. In addition to high hardness, diamond also has high chemical and mechanical resistance, high thermal conductivity, and good electrical insulating properties, so it is mainly used to produce grinding, cutting, or drilling tools. However, it has also found its application in medicine, for example, as part of orthopedic instruments where, in the form of nanostructured coatings, the effect can be more pronounced when the layer is further modified [13], it reduces product friction, protects against wear, and thus prolongs product life; in the form of a nanodiamond, due to the small particle size, purity and functionality to functionalize their surface, for targeted drug release [14]; or as part of microfluidic devices [15].

The attractiveness of diamond thin films extends to exotic areas of superconductivity and quantum computation through controlled thin film doping. As a result, diamond is one of the most sought-after materials with many applications, from abrasives, protective coatings and biomedical applications to high-end diamond electronics, photonics, and imaging equipment [16,17,18,19].

**Figure 2:**
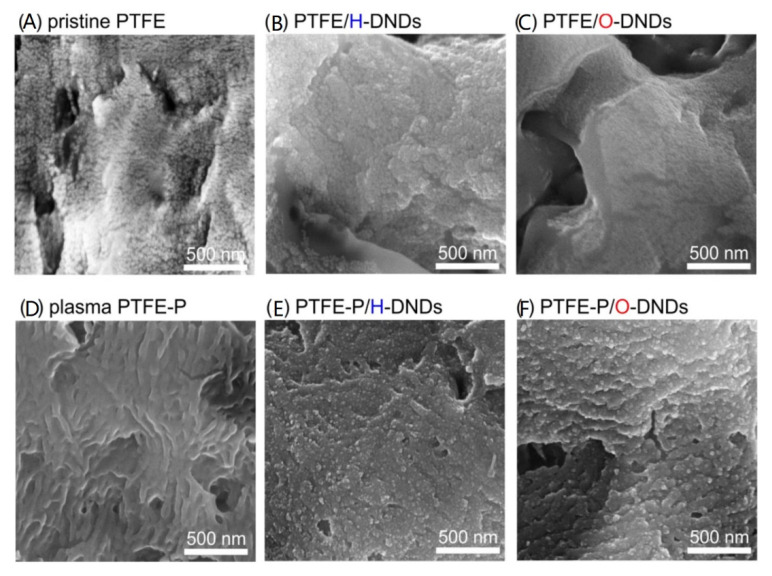
Scanning electron microscopy images (secondary electrons). SEM images of pristine PTFE foil (**A**) before treatment; (**B**) after hydrogenated nanodiamond treatment; and (**C**) after oxidized nanodiamond treatment. SEM images of the plasma-treated PTFE foil (**D**) before treatment; (**E**) after hydrogenated nanodiamond treatment; and (**F**) after oxidized nanodiamond treatment. Reprinted with the permission from ref. [19], 2018, John Wiley and Sons. DND refers to detonation nanodiamonds (DNDs).

Due to its remarkable properties, nanodiamond is very suitable for composite materials and thus improves the properties of other materials (Figure 2). In this case, the nanodiamond acts as a filler, the matrix being formed by a polymer or other inorganic compound. The nanodiamond can be bound in composites by both covalent and non-covalent bonds [10]. Composite materials containing nanodiamonds are commonly used as coatings. Stainless steels with composite coatings containing apatite and nanodiamonds (with a size of nanodiamonds 4–6 nm) have been shown to significantly improve adhesion, ductility, metabolic activity, and hardness. Their biological activity has also been demonstrated [20]. Another material that has not been extensively researched and described is carbon-carbon composite. It can be, for example, a composite called “nanostructured diamond” (NSD) or “ultrafine nanostructured diamond” (USND), which is composed of nanocrystalline diamond grains 5–6 nm in size, which are embedded in an amorphous carbon matrix [21].

### 2.3. Graphene

Graphene is a unique transparent material with a high specific surface area [22], excellent thermal conductivity, quantum Hall effect, good absorption (from visible to near IR) and optical transparency of approximately 97.7% [23], high carrier mobility, and high Young’s modulus [22], etc. It consists of a planar network of one layer of carbon atoms arranged in the shape of hexagons connected by bonds. Thus, the only fundamental unit of graphene is carbon. Ideally, it is a monolayer of sp2 hybridized carbon atoms with a hexagonal lattice (one layer of graphite). Each carbon atom has three σ-bonds and a p-bond outside the plane that can bind to adjacent atoms [24,25]. This atomic structure, combined with the electron distribution of graphene, leads to high thermal and electrical conductivity, unique optical behavior, excellent mechanical properties, extreme chemical stability, and a large surface area [25,26]. Graphene may not only exist in a flat two-dimensional (2D) form but, depending on the physical dimensions, different types of waviness can be defined, such as ripples, wrinkles, folds, or creasing of graphene [27]. Graphene can be transformed into graphene materials by chemical and physical modifications, such as single-layer and multilayer graphene, graphene oxide, and reduced graphene oxide, each of which has unique properties. By scrolling graphene, carbon nanotubes or nanohorns can be obtained [25,28]. All these properties enable sufficiently efficient graphene for universal applications in electrochemical devices [29] and other applications [30].

Graphene is widely used in biomedicine as a basis for cell growth, mainly due to its biocompatibility, chemical interactions, flexibility, and electrical conductivity. Due to the possibility of modification and functionalization of its structure, it serves in tissue engineering as a bioactive scaffold, which can bind the necessary proteins or drugs [2]. The excellent mechanical strength and stiffness of graphene materials are suitable for bone tissue engineering. The good electrical conductivity of graphene materials is, in turn, suitable for nerve tissue engineering [31,32,33]. Due to the possibility of modification and functionalization of its structure, it serves in tissue engineering as a bioactive scaffold and can bind the necessary proteins or drugs [2,34,35,36]. Excellent mechanical and thermal properties also characterize graphene. A complication of working with graphene is its poor dispersion in solvents and aggregation [37].

Graphene is very often used in combination with carbon fibers and polymeric materials. One type of such composite is layered materials, where a graphene-epoxy composite is used to make a prepreg having the form of carbon fabric. This prepreg is then used for interleaving into a carbon fiber reinforced composite [38]. In this case, the graphene plate has the function of a curing filler. The combination of graphene sheets and epoxies leads to a significant increase in the fracture toughness and/or fatigue life of graphene/epoxy interleaves for delamination toughening and the monitoring of crack damage in carbon fibre/epoxy composite laminates [39]. Graphene in the form of nanoplates embedded in a PLLA type polymer causes a decrease in the electrical resistance of the material in samples with 20 wt% of graphene nanopellets; in volume, the value of electrical resistance decreases down to 150 Ω. In this study, the positive effect of nanopellets in polymeric material on cell adhesion and proliferation has been shown [40].

**Figure 3:**
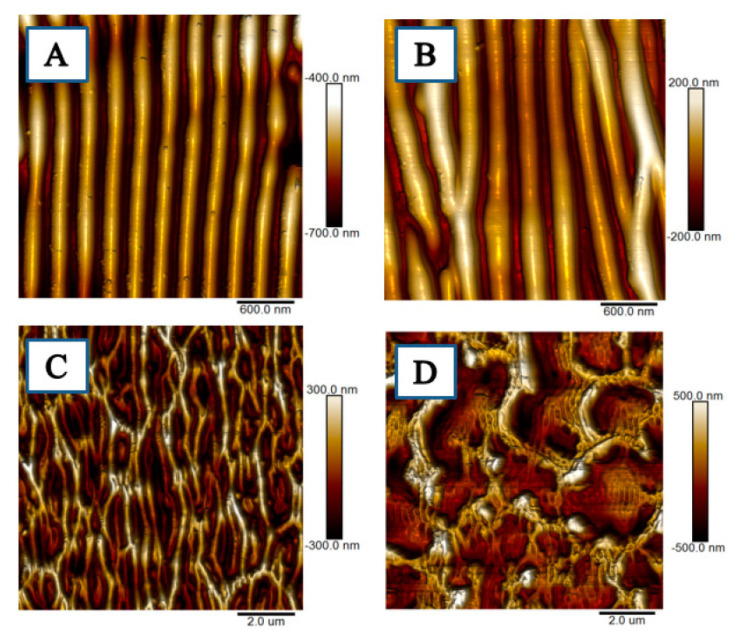
AFM scans of pristine polystyrene modified with 6000 pulses and laser fluence values: (**A**) 8, (**B**) 10, (**C**) 12, and (**D**) 16 mJ∙cm^−2^ Reprinted from ref. [43].

Composite films can also be prepared by combining graphene with other functional nanomaterials (precious metals, metal compounds etc.). These then show unique optical, mechanical, electrical, chemical, sensing, and catalytic properties [41]. The combination of graphene and, e.g., ZnO, increases the photoactivity of such a composite [42]. Graphene composites with polymers can also be further treated with the aim of creating periodic or other regular structures (Figure 3) [43]; the pristine polymer may also be activated by excimer laser [44,45,46,47] for subsequent carbon deposition.

Graphene (but only in its transparent form) is very often studied for its potential use in photovoltaics, optoelectronics, and photodegradation and photodetection devices, etc., especially in combination with polymers such as epoxies, polystyrene, polyaniline, polyurethane, polycarbonate, and PET. Here they can find a use as part of reliable super flexible perovskite solar cells or as anodes in solar cells. Graphene is also used as an effective conductive filler to produce composite structures for EMI shielding [48,49]—again, in combination with polymeric materials.

The use of graphene and graphene-based materials in composite applications is limited mainly by poor solubility and dispersibility in liquids. However, the excellent interfacial adhesion is also a crucial parameter for composite materials [50]. The simplest dispersion method is mixing or shear mixing of a colloidal graphene suspension with a selected polymer [51]. Sonication without/with surfactants is often used. Unfortunately, this is an unsatisfactory method for many applications because the presence of a surfactant can affect the transparency, thermal properties, and mechanical properties of the composite [50,51,52]. Superacids have been shown to be a suitable solvent for graphene, in which the graphene concentration can be up to 2 mg/mL. Again, emphasis must be placed on potential applications, as superacids are usually incompatible with most composite applications. The oxidation of graphite to form graphene oxide is also a widely used technique for dispersing graphene in the liquid phase [52]. The in situ polymerization technique (involving mixing the filler in pure monomer/multiple monomers or in a monomer solution followed by polymerization in the presence of a dispersed filler) has proven to be a very suitable method for forming composites with well-dispersed graphene [51]. In situ polymerization, in which graphene is “coated” with a polymer coating, is also used to prevent aggregation due to Van der Waals forces. It combines emulsion and micellar polymerization because the graphene is inside the micelle, and a thin polymer coating is applied to the graphene surface. This method has proved successful in forming reinforced polymer composites, where polyvinylpyrrolidone has been used for coating. Such materials can then be used to make mechanically strong and electrically conductive graphene/PVP composites [50,52]. The preparation of graphene from graphene oxide (GO) is carried out by reduction, leading to many defects. The graphene thus prepared is referred to as chemically modified (reduced) graphene (rGO). Both GO and rGO start from the same material, but structure and surface characteristics can vary significantly depending on how the GO is peeled off and reduced. Oxidation of graphite and thermal or chemical reduction leads to irreversible deformation of graphitic carbons, which can modify micromechanical and electrical transport properties. GO is an electrical insulator, so it is necessary to reduce it to restore electrical conductivity and other properties belonging to graphene. Surfactants can be used in this case to prevent aggregation [2,53,54].

The structure of graphene is the starting point for the formation of other carbon allotropic modifications, such as fullerenes and carbon nanotubes [55].

### 2.4. Carbon and Graphene Quantum Dots

Carbon quantum dots (CQDs) are one of the most recently discovered fluorescent nanomaterials. They usually contain amorphous or nanocrystalline nuclei with predominantly sp2 hybridized carbon. CQDs are nano-dimensional objects. The usual size of these objects is below 20 nm, and most often they are semiconductors in which the so-called quantum trapping of a particle occurs. The surface structures of CQDs vary widely depending on the preparation methods and the precursors used for the synthesis, and they may contain oxygen/nitrogen, functional groups. If the dimensions of the material are large compared to the wavelength of the electrons inside, then these electrons can move as free particles. However, once the size of the material is reduced and its dimensions are comparable to the wavelength of the electron, then the optical and electrical properties of the material will differ from those of the large bulk material. With the decreasing dimensions of the material and when reaching a specific optimal limit, called the Bohr radius of the exciton, its energy spectrum will become discrete, and the width of the bandgap will depend on its dimensions. The most significant consequence of this phenomenon is the confinement of the electron to a smaller space than the de Broglie wavelength of the electron. Due to quantum confinement, the material’s band structure is changed, as is the thickness of energy levels. If the material is a semiconductor, then changes in the density of energy levels in the conduction and valence bands will affect the occupancy density, the transitions between the bands, and the mobility of the charge carriers. The size of the forbidden EG band will be larger than the EGB “bulk” of larger-sized material, and the optical and electrical properties will also change [56].

Graphene quantum dots (GQDs) usually have a diameter below 20 nm. Compared to commonly used inorganic QDs (e.g., CdSe), characterized by their colloidal behavior, limiting their stability in many applications, GQDs have the advantage of non-toxic behavior and easy handling [37].

The possibilities of preparing GQDs can be divided into two main categories: the so-called top-down and bottom-up methods. Top-down methods involve the decomposition and exfoliation of cheap and readily available material, most commonly graphite, under “harsh” conditions. These methods often require multi-step operations, most using acids, potent oxidizing agents, and high temperatures. However, their disadvantage is the small control over the morphology and size distribution of the formed particles. Bottom-up methods involve the synthesis of QDs from polycyclic aromatic compounds, such as fullerenes. This approach allows one to control the final properties of the resulting product [37].

Due to their properties, GQDs have also found use in medicine, for example, as a tool for so-called photodynamic therapy in the treatment of cancer, which has recently replaced commonly used methods such as surgery, chemotherapy, and radiotherapy, mainly due to minimal side effects and negligible drug resistance and low systemic toxicity. GQDs and functionalized GQDs have a high potential for use in biotechnologies due to their optical properties, but mainly due to their low cytotoxicity [57,58,59,60].

Graphene Quantum Dots are fluorescent substances that retain the structural and physical properties of graphene oxide. Their connection with hydrogels leads to an expansion of the possibilities of their potential applications in medicine. If GQDs are used in the form of nanoparticles placed on a carboxymethylcellulose hydrogel, the tested properties will be improved. Such modified hydrogels have the potential to function as a long-lasting and highly effective anti-cancer agent [61,62].

### 2.5. Fullerenes

Fullerenes, sometimes called Buckminsterfullerenes, represent a carbon allotropic structure that consists of an even number of sp2 hybridized carbon atoms. These are connected into 12 pentagonal and m hexagonal rings where m = (n − 20)/2, where n represents the total number of carbon atoms in the molecule. C60 (12 pentagons and 20 hexagons) is thus the smallest fullerene that satisfies this so-called pentagon rule, which states that the most stable are those fullerenes where pentagons do not share any edges [63]. Several studies have been performed showing that the stability of fullerenes increases with their size [64,65]. Due to the curvature of the structure, fullerenes undergo so-called pyramidalization, i.e., a change in hybridization from pure sp2 to an intermediate between sp2 and sp3 hybridization. These changes affect the resulting properties of fullerenes, causing, for example, high electron affinity or an increase in chemical reactivity, and thus fullerenes are more subject to additive reactions. Thanks to these specific properties, fullerenes have found application across the chemical and medical industries. They are used as systems for targeted drug delivery (drug delivery system), nanosensors, antioxidants, or as construction materials for solar panels [66]. Due to their high reactivity, they can be used as catalysts; their chemical functionalization (combination with other compounds) allows the creation of various combinations of unique properties [1] and also allows a significant increase in the solubility of fullerenes in many solvents because “pure” fullerenes are minimally or not insoluble. Many other potential applications of fullerenes are superconductors, lubricants, photovoltaics, sensors, imaging technology (using photoluminescence of fullerenes), etc. [67,68,69].

Fullerenes have many unique properties, e.g., the ability to withstand high temperatures and capture smaller substances such as helium [67], and they show a wide range of biological activity. For example, fullerenes can release electrons and convert molecular oxygen to singlet—atomic oxygen when irradiated with UV radiation. As a result, they can cause damage to cell membranes and cleave DNA, which can be used in photodynamic therapy to treat tumors or against antibiotic-resistant bacteria [70,71].

**Figure 4:**
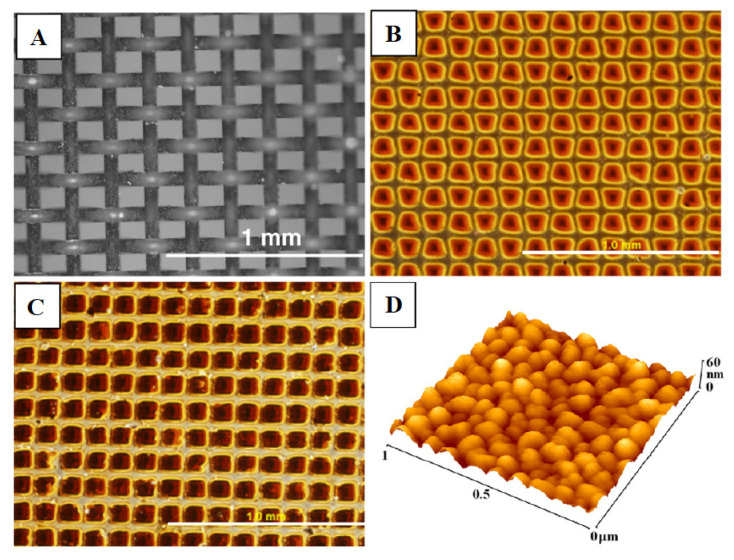
Morphology metallic grid used for creating micropatterned C60 layers (**A**) and morphology of thin (**B**,**D**) and thick (**C**) micropatterned C60 layers (D: on a bulge). Olympus IX 50 microscope equipped with DP 70 digital camera and atomic force microscope Digital Instruments CP II Veeco. Reprinted with the permission from ref. [7], 2009, Elsevier.

In another study [7,67] it was found that fullerenes, when arranged in thin layers, can be used as a nanostructured material (Figure 4) and positively affect the adhesion, spreading, growth, and viability of bone cells, specifically human osteoblasts-like MG 63 cells.

Therefore, fullerenes are often considered a tool and basis for creating promising materials with new properties. Fullerenes can be used as reinforcing agents for light metal matrix composites. These materials then have excellent mechanical properties and high chemical stability [72,73,74]. Metal-fullerene materials improve a wide range of physical and physico-chemical properties. The addition of fullerenes to the materials, even in small amounts (up to 1.0% by weight), significantly changes the properties of the original material [75]. These composites are mostly used to produce active elements for sensors, in the field of nano- and micromechanics, or for coatings, and not only in the area of biomedical applications [76].

Another interesting material with potential promising field-emission properties is created by a combination of fullerenes and single-walled nanotubes. It is a hybrid material created during a continuous process. In this synthesis, fullerenes [77] are covalently attached to the outer surface of a single-walled nanotube.

### 2.6. Carbon Nanotubes and Nanofibers

**Figure 5:**
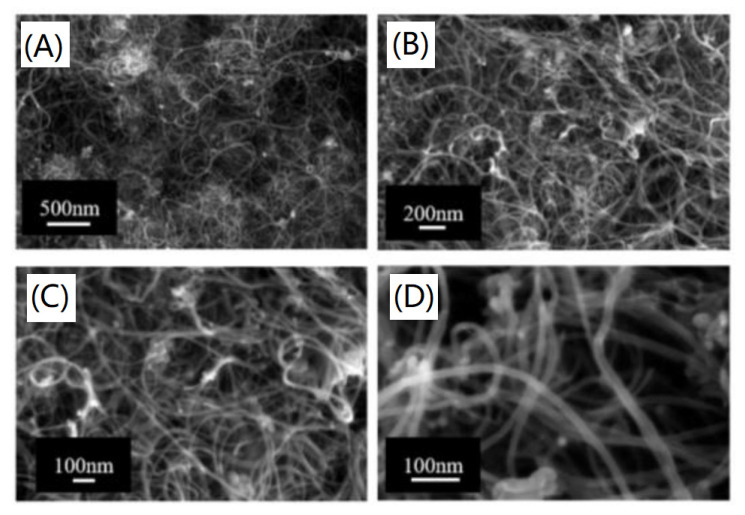
FESEM images of MWCNTs at different magnitudes:(**A**) 70 kX, (**B**) 130 kX, (**C**) 250 kX, (**D**) 500 kX. Reprinted from ref. [78].

Carbon nanotubes (CNTs) may be considered to have a simple atomic configuration: graphene sheets rolled into cylindrical shapes. Depending on the number of graphene layers, they are divided into single-walled nanotubes (SWCNTs) and multi-walled nanotubes (MWCNTs) (Figure 5) [78]. While SWCNTs contain only one layer of graphene, MWCNTs consist of nested cylinders with a layer spacing of 3.4 Å and a diameter in the order of 10–20 nm. CNTs further differ depending on how the graphene sheet is rolled into “zigzag”, “chair”, or “chiral” nanotubes [79,80,81]. The optimal process for surface enhancement, connected with wettability and surface chemistry changes, is plasma exposure of the polymer surface [82,83,84,85], which can be further used for subsequent nanoparticle deposition or grafting.

Carbon nanofiber (CNF) is formed when graphene sheets are curved at a certain angle (α) and thus create a stack of nanocones. CNF differs from carbon nanotubes (CNTs) by an angle, α, which is equal to zero for CNTs. The size of the CNF can be from 3.5 nm to several hundred nanometers in diameter, with a length of up to several micrometers [86]. The small diameter significantly affects the number of structural defects that affect the surface’s mechanical properties and specific properties. Carbon fibers can have a different structures: hollow- and filled-core, stacked nanocones; partitioned, stacked nanocones; and partitioned nanotubes [81]. Carbon nanofibers can be used to treat and stimulate the bone system and the construction of nanocomposite or fibrous substrates in situ tissue engineering. Their biocompatibility has been confirmed in many studies [87,88,89]. CNTs and CNFs can be used for biosensor generation [90] or nerve tissue engineering [91]. Carbon fibers are very light materials with a low density of 1.6–2.2 g∙cm^3^ [92,93] and excellent mechanical properties, which predestines them for use in high-performance composites, especially in the aerospace industry [93,94,95].

Single-fiber and multilayer fibers and single-walled and multi-walled tubes have many unique properties that make them suitable for use in composites. However, as in other carbon materials, the primary intention is to use them to improve the properties of the original, mainly polymeric, ceramic, and to a lesser extent, other inorganic materials (metals such as Al_2_O_3_).

Both types of carbon materials (fibers and tubes) have a very low density in the case of fibers; their diameter is significantly smaller than their length. This makes them ideal candidates for composite fillings in the form of reinforcements [10,96]. The biggest problem in forming these types of composites is the homogeneous distribution of carbon nanoparticles in the matrix. This can further lead to the aggregation of nanoparticles and the formation of sites with different filler concentrations, causing an inhomogeneous distribution of properties. One important parameter is also the arrangement of carbon fibers or tubes in the matrix. The direction of arrangement of the fibers and tubes can affect, for example, the load-bearing capacity of the polymer composite [10].

When carbon tubes are combined with suitable polymers—such as poly (p-phenylene vinylene) or its derivatives, the prepared composites have electroluminescent properties and are used in photovoltaic devices and light-emitting diodes. In combination with other polymeric substances, the electrical properties are significantly improved. In these cases, the nanotubes were used in combination with inexpensive polymers and served as a replacement for the still frequently used carbon blacks. They had the advantage over carbon black in that the electrostatic charge was reduced when nanotubes were used. In another study, it was found that adding 1% by weight of carbon nanotubes to a polystyrene matrix increases elastic stiffness by about 36–42% and increases tensile strength by 25% [10]. The use of carbon fibers in combination with phenolic prepregs containing nanoparticles resulted in the production of refractory material [97].

However, nanofibers and nanotubes can also be used in conjunction with other materials [98]. For example, when properly combined with ceramic materials, composites with excellent temperature stability and resistance, excellent toughness, and creep resistance could be formed. In the case of combination with metallic materials, an improvement in the electrical conductivity of aluminum has been demonstrated. This increase is due to an increase of the nanotubes content in composites [97]. Carbon fibers in the matrix made of aluminum or magnesium are used today in some areas [99,100], where it is necessary to ensure low density and high strength of the material, such as in the aerospace industry [101]. The carbon fiber and tube composites used today can be found in a wide range of applications. They are part of, e.g., baseball bats, golf clubs, car parts (not only fuel pipes) or damask steel [102].

As in graphene, the potential use of CNTs is limited due to difficult dispersion and poor interfacial interaction, especially with the polymer matrix. In the case of CNTs, the situation is more complicated because CNTs are characterized by a small diameter on a nanometer scale with a high aspect ratio (>1000) and thus an extensive area [103]. CNTs are delivered commercially as strongly entangled bundles, so adjusting the “cut and unwind or unwind” before the dispersion itself is necessary to activating of treatment [104]. Sonication is used as the most common method. Ultrasound is suitable for the dispersion [105] of CNTs in low density liquids. CNTs can also use surfactants (dispersants), which are usually added to the polymer melt [103,106]. Ball milling in the presence of various chemicals can also be used, which thus leads to the introduction of functional groups on the CNT surface. In the case of CNTs, surface functionalization can also be used through carbon atoms located both on the ends of the tubes and on the walls [105]. Defects in which defect sites are transformed can also be functionalized [103,104,107]. While -COOH and -OH are highly polar, the amide groups (-CO-NH-) and non-polaroctadecyl groups (-C18H37) make CNT-ODA highly stable in mixed polar-nonpolar (butanol-xylene) solvents. The presence of these groups increased the tensile strength of functionalized CNTs by 4–7% (depending on the type of group [107]. Functionalization can also be performed by activating, e.g., carboxyl groups, and hydroxyl groups can be formed on the surface during the oxidation process by oxygen, air, concentrated sulfuric acid, nitric acid and 30% aqueous hydrogen peroxide, and concentrated sulfuric acid and its mixture [104]. Active aldehyde (–CHO) and amino (–NH_2_) groups can be introduced to the surface by plasma modification in the presence of a pair of acetaldehyde and ethylenediamine [104]. When CNTs and polymer resins are combined, the chemical functionalization of the CNTs prevents the CNTs from interacting with the adjacent CNTs in the resin due to the cross-covalent bonds between the CNTs and the surrounding polymer. This will lead to a better quality of the CNT dispersion in the resin [108].

Fibrous composites are used due to their exceptional mechanical properties, impact resistance, high durability and flexibility in design capabilities, and low weight. The problem with fiber-predominant composites is delamination (cracks between layers), limiting the life of the material. One solution is to incorporate some stiffening agents or thermoplastic binders into the brittle matrix resins. Another possible solution is to functionalize the surface of CNTs. Functionalization of double-walled carbon nanotubes with amino groups increased the fracture toughness value by 42%. In the case of glass fiber/epoxy fibrous materials, the strength can be improved (by 16%) by simply adding a small amount (0.3 wt%) of double-walled carbon nanotubes [106]. The mechanical strength of the polymer/CNT compound can be significantly increased by periodically patterning the surface of the carbon nanoparticles (cheetah skin) [105]. A good ratio between toughness and strength in polymer composites containing CNTs can be obtained by chemical functionalization of said fillers. Several studies reported in [109] report pimelic acid or Calcium pimelate as a suitable chemical compound. In these studies, it has been shown that chemical functionalization leads to increased toughness and ductility of composites and improved compatibility between the filler and the polymer matrix. Carbon nanofibre composite with silicon was reported as a suitable anode material for lithium-ion batteries [110].

### 2.7. Carbon Nano-Onions

Newer carbon nanostructures include carbon nano-onions (CNOs), also known as multilayer fullerenes. They are such a combination of fullerenes and multi-walled nanotubes. They can also be considered as so-called fullerene cages. There are two types of multilayer graphitic objects: real carbon onions, showing a concentric structure of spherical shells, and bulbous graphite nanoparticles, with a strongly faceted shape and a significantly larger internal cavity. Their structure contains hexagonal and pentagonal rings with carbon atoms located at the vertices. They form two single bonds and one double bond with adjacent carbon atoms with delocalized π-electrons. The graphitic layers in the structure are composed of many defects and holes, which can be filled in various ways with heptagonal and pentagonal carbon rings. This produces an amorphous or crystalline quasi-spherical carbon nanocell [111]. The classical size of carbon nanotubes is 4–25 nm [112]. These structures are often used as high-speed anodes. Still, their electrical properties depend on the conditions of their synthesis—they can be prepared by a variety of methods such as CVD, vacuum annealing, arc discharges, etc. [113,114,115]. In vitro and in vivo experiments have shown promising properties, including low cytotoxicity and low inflammatory potential, confirming the ability of CNOs to be used as imaging agents and for targeted drug delivery [116,117].

Carbon nanocells have many structural defects. This, together with their curved shape, can be used for conversion to other forms of carbon, specifically to particles with a diamond structure. In addition to irradiation with high-energy electrons, the transformation of a carbon onion to a diamond also succeeds by bombarding with ions such as Ne^+^, thermal crumbling at temperatures of 500 °C, or by irradiation with a CO_2_ laser.

### 2.8. Globular Carbon Nanoparticles

**Figure 6:**
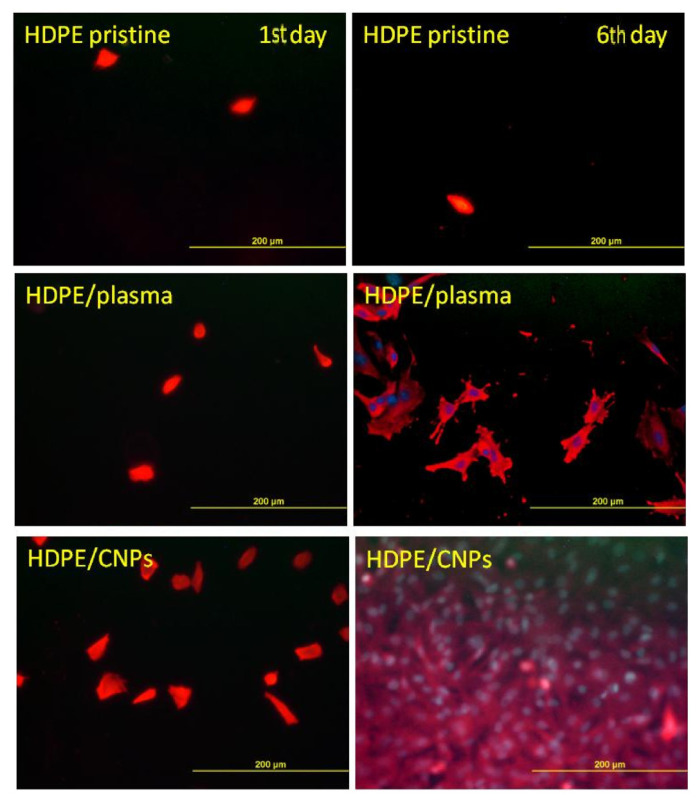
Fluorescence microscopy images of VSMCs adhered (1st day) and proliferated (6th day) on pristine (HDPE) and CNPs grafted (/CNPs). Plasma exposure time for all modified samples was 120 s Reprinted with the permission from ref. [123], 2017, Elsevier.

Activated carbon, charcoal, and carbon black have a high specific surface due to their particle structure. Small and irregularly arranged graphitic domains make the material practically isotropic. In general, there are two types of carbon black—carbon black as a product of incomplete combustion and carbon black obtained by thermal decomposition [10]. Carbon nanoparticles could, due to their leaked properties, be used in a wide range of applications. However, in most cases, they have some serious drawbacks that preclude them for use in particular applications. For carbon-based materials, and especially nanoparticles, it is often their tendency to aggregate. Methods of surface functionalization can be used to reduce aggregation or its complete elimination, especially with the help of hydrophilic groups [118]. The functionalization itself can be performed in different ways using different substances. Metal nanoparticles are most often used, but various chemical groups or other substances can also be chemically attached to the surface of nanoparticles. Some studies [119,120] show that the introduction of a nitrogen, sulfur, or boron heteroatom can positively affect the properties of carbon nanoparticles; for example, nitrogen can be used in various forms—such as nitro or amino [121]. It has been found that amines that have a positive charge can be used to increase the attractiveness of carbon nanoparticles in the study of cell interaction [122]. Such functionalized nanoparticles can be used, either alone or bonded to another type of material. In tissue engineering, polymeric materials are very often used, and they could be modified in this way and potentially used, for example, for cell growth and culture (Figure 6). The combination of a suitable substrate and a subsequently functionalized nanoparticle would take advantage of the properties of the polymeric material and at the same time improve its shortcomings (low bio- or cytocompatibility) [123].

### 2.9. Q-Carbon

**Figure 7:**
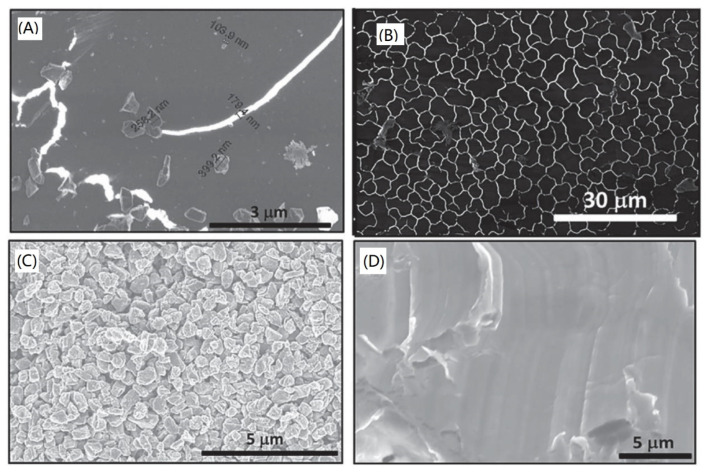
(**A**) Formation of Q-carbon and growth of diamond from Q-carbon, (**B**) large-area Q-carbon, (**C**) microcrystals of diamond, and (**D**) large-area single-crystal diamond film on (0001) sapphire. Reprinted from ref. [130].

However, attention is currently focused on a new carbon phase, called hardened carbon (Q-carbon, Q-C), which exhibits excellent ferroelectric properties, even better than diamond [124]. Q-carbon has unique mechanical, chemical, and physical properties, thanks to which it has been given high attention, especially in field-controlled electronics and biomedicine [125,126]. Q-carbon has an amorphous structure consisting of 75–85% sp3 and residual sp2 hybridized carbon atoms, leading to various exciting properties such as extraordinary Hall effect, field emission, and higher hardness than diamond [127,128]. Most interesting of all, Q-carbon exhibits ferromagnetism at and above room temperature [129]. When assessing the hardness of a material, it is necessary to focus on its response to the action of external forces. Under the influence of an external force, the material undergoes a reversible elastic or irreversible plastic deformation. Q-carbon, consisting of a tightly arranged tetrahedral structure, allows one to increase the density of the number of particles by up to 50% in crystalline and up to 60% in a random arrangement and increase the hardness by 40% compared to diamond. Q-carbon, discovered in 2015, is a recently discovered metastable carbon phase (Figure 7) [130]. It is formed by transforming thin layers of amorphous carbon by nanosecond pulsed laser melting and subsequent hardening in the subcooled state [131]. Q-carbon can be prepared by melting with a nanosecond pulsed laser (PLA) followed by ultrafast quenching of amorphous carbon. During the nonequilibrium process of pulsed laser annealing, carbon melts. This melt can then be converted into various carbon polymorphs. The second ultrafast hardening step causes the molten state to remain below the equilibrium melting point due to subcooling. If the subcooling is large enough, metastable states (diamond and Q-carbon) with a lower melting point than the stable graphite phase can nucleate. The size of the subcooling thus determines the resulting phase formed and the kinetics and growth rate [132].

It has been suggested that liquid carbon may exist as a thermodynamically stable phase at high pressures and temperatures. Depending on the energy density of the laser and the physical properties of the amorphous film and substrate, a process of supercooling and quenching occurs, leading to a metastable state of the amorphous Q-carbon structure with unique properties [133]. It has been shown that nanosecond laser heating of diamond amorphous carbon on sapphire, glass, and polymer substrates can be reduced to the melting of carbon in a very subcooled state [129]. Based on experiments and simulations of the interaction of a laser with a solid film, it has been found that subcooling allows the formation of Q-carbon when growth rates are 6–16 m∙s^−^^1^, while speeds of 4–6 m∙s^−^^1^ are sufficient for diamond formation [132]. Q-carbon plays a crucial role in diamond formation—nanodiamonds, microdiamonds, microneedles, and single crystal thin films can be formed depending on the allowed conditions of nucleation and growth for diamond formation [129,134].

Due to the high hardness of Q-carbon mentioned above, this material is suitable as a part of a composite, where it ensures strength and hardness as a reinforcing component in a softer matrix. Protective coatings for tribological applications must meet three primary conditions: hardness, strength, and satisfactory adhesion to the substrate. Although diamond and DLC layers show excellent hardness, low coefficient of friction, and biocompatibility, the problem may be their lower adhesion and thus shortened service life due to peeling. Furthermore, the action of a pulsed laser on the carbon film on the substrate creates compressive stress at the film/substrate interface, which results in tensile deformation in the upper layers. This results in the formation of filamentary Q-carbon, providing hardness and strength under the α-carbon layer, which acts as a matrix of the composite, ensuring softness and adhesion [135]. The effect of the nanocomposite Q-carbon coating has been investigated on stainless steels, which are used in the chemical, petrochemical, and medical industries. Coatings on stainless austenitic steels for medical use primarily fulfill a protective function, preventing direct contact of steel with body fluids and preventing its corrosion and releasing iron, chromium, and nickel ions into the body [136].

### 2.10. Carbon Aerogel and Aeroraphite

In recent years, research regarding energy applications has focused on carbon aerogels. Due to their high electrical conductivity, mechanical strength, low density, large area, and well-controllable structure, they can be used as absorbents, catalysts for hydrogen fuel storage or supercapacitors, electrodes, or as insulating materials [137,138,139]. Carbon aerogels are 3D nanogrids with an open porous structure that allows the penetration of molecules and ions into the interior space [140]. The microstructure consists of interconnected solid phase particles—carbon grains, in which there is a network of interwoven graphite fibers with a width of several units (nm) [141], and it is the optional and customizable porosity that makes them excellent candidates for extreme applications such as space vehicles [142]. Carbon aerogels also excel in extremely high heat resistance and stability. They can retain their porous structure in an inert atmosphere, even at very high temperatures of 2273 K [139]. There are three main approaches to aerogel preparation: (i) direct carbonization of aerogels, (ii) self-assembly of nanocarbon building blocks, and (iii) chemical vapor deposition. However, production is accompanied by severe shortcomings (nanostructure often shrinks and collapses during the carbonization process, very expensive and often toxic precursors are used for production, complex and costly equipment is required for production), which hinders the practical development of carbon aerogels [140,142].

Graphene-based aerogels have been shown to have high adsorption performance and excellent recyclability of oils and organic solvents. However, acid waste is produced during the synthesis, which severely limits industrial production. Therefore, there is a need to develop a simple, economical, and efficient method to synthesize a carbon-based aerogel on a large scale. In recent years, carbon nanofiber aerogels with good oil absorption and organic solvent properties have been prepared from synthetic bacterial cellulose. However, their disadvantages, such as hydrophobicity, low adsorption capacity, poor buoyancy, and unsatisfactory recyclability, prevent further application in oil water separation. These disadvantages have been overcome by the use of another organic material, namely kapok fiber. A light, hydrophobic porous carbon microtube aerogel (CMA), which has a high absorption capacity with various organic solvents and oils, can be prepared from this fiber by a straightforward method. Its CMA adsorption capacity reached 78–348 times its weight. CMA also has excellent thermal stability, and thanks to its excellent mechanical properties, CMA can be recycled by distillation, pressing, and incineration. The prepared CMA is therefore widely used as an economical and safe adsorbent for environmental protection. In addition, CMA can be used as a 3D electrode material for energy storage or as a building block for other functional composites [143].

An exciting alternative to aerogels are materials called aerographite. These are entirely black, optically opaque materials with a very low density (<200 μg·cm^−^^3^) and extreme robustness capable of withstanding strong deformations and remarkable mechanical tensile loads without losing structural integrity. Techniques have already been developed to prepare aerogels in various macroscopic shapes of several cm^2^. The porous structure of aerographite is made of an interconnected network of microtubes with a nanoscopic thickness (≈15 nm). Depending on the preparation technique used, a number of variants of aerographite can be created, other types of carbon can be inserted into hollow microtubes, and it is possible to tune the morphology of aerographite in a broad sense about its aspect ratios, diameters, and surface structure. The CVD method can be used to prepare aero-graphite. By one-step synthesis using ZnO templates, materials of the order of cm^3^ can be prepared with excellent mechanical resistance, specific stiffness, and tensile strength, and an extremely low Poisson’s ratio. The atomic structure can be tuned from graphitic to glassy pyrolytic carbon. Properties such as superhydrophobicity (excellent wetting with epoxy systems for the production of nanocomposites), conductivity, flexibility and compressibility without loss of structural integrity, high optical absorption and X-ray opacity, high temperature stability and chemical resistance, tensile and compressive strength, and superhydrophobicity it makes it a remarkable multifunctional material. Potential applications for these materials can be found in areas such as microelectromechanical systems (MEMS), electrical shielding, or the application of conductive electrodes, which should withstand high accelerations, for example, caused by vibrations or shocks [144].

### 2.11. Carbon-Carbon Materials

A special group of composite materials is carbon-carbon materials. These are composite carbon matrices reinforced with carbon fibers or nanoparticles. The advantage of such materials is their low density (usually 1.6–2 g∙cm^−^^3^) compared to metal or ceramic composites [145].

Their other advantages include a high strength-to-weight ratio, high resistance to thermal shocks, and chemical stability. These are very specific combinations of carbonaceous materials, so their use is also very specific. These materials have been used, for example, in the production of nozzles for rocket engines and for aircraft brakes. However, their big problem is oxidation at temperatures exceeding 773 K, when their strength decreases. This limits their use only in an inert atmosphere. One possible solution to this problem is to coat the composites with silicon carbide (SiC) [146].

### 2.12. Carbon Materials Prepared from CO_2_

Carbons converted to CO_2_ may become promising candidates for high-capacity, high-speed, low-cost, and large-area electrode materials in future supercapacitors. Indeed, the preparation of various carbon materials based on CO_2_ conversion technology has been intensively studied in recent years and has already been recognized as an advanced strategy for the green construction of electrode materials with promising application prospects. Selective reduction of CO_2_ to pure carbon is expected to be a good alternative for greenhouse gas recycling; however, the high stability of the C = O bond in CO_2_ makes it a significant problem. Chen Li et al. [147] state that several carbonaceous materials (CNT, CNF, GO, etc.) can potentially be used as nanostructures for energy storage, fuel, and articles by providing backup power and the prevention of power outages that serve as essential accessories for military purposes, electric vehicles, intelligent tools, and portable electronic devices. Various methods have been used for this, including direct metallothermal combustion, carbonate conversion, high temperature reduction, and controllable self-sufficiency reactions. Each method has its advantages and disadvantages regarding preparation efficiency, energy consumption, product quality, etc. Therefore, the details of the technology should always make appropriate improvements to address these issues [147].

### 2.13. Diamond-Like Carbon (DLC) and PVD Technique

**Figure 8:**
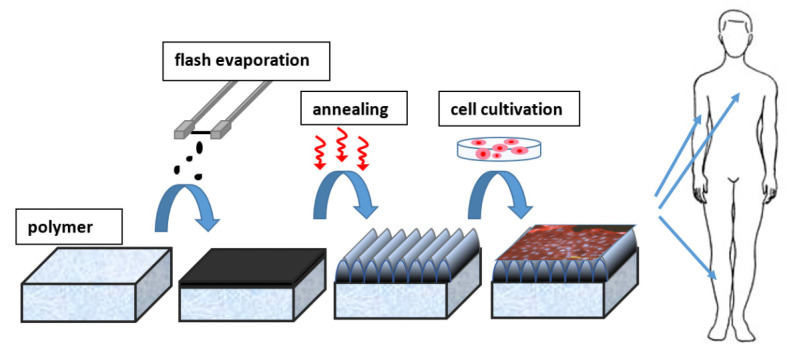
Scheme of application of carbon coated LIPSS in tissue engineering Reprinted with the permission from ref. [151], 2019, Elsevier.

Carbon is used daily, not only in its natural form, in the form of nanoparticles, but also in the form of layers or nanolayers. It should be emphasized that it is very difficult to precisely define the thicknesses of the layer below which the layer becomes thin. This is mainly because, with the thickness of the layer, the properties change differently. In general, however, the value of the thin film thickness has stabilized below one μm, deposited by one of the following methods. Another way the boundary between a thin layer and a thick layer is defined is whether the layer has surface or “bulk” properties [148,149]. The application of thin films strongly depends on the selected material’s electrical, optical, and physical properties. On the contrary, these properties depend on the selected technique and the process of thin film preparation. Using the same process and preparation method can lead to the formation of a transparent, dielectric film with excellent optical properties, while by slightly adjusting the set parameters, an opaque film suitable for electrically conductive rather than optical applications can be prepared. Thin films thus play an important role in a wide range of industrial and medical applications (Figure 8) [150].

Diamond-like carbon (DLC) is a group of obscure carbon materials with a unique diamond property. It is a metastable form of amorphous carbon containing a significant proportion of sp3 bonds (a mixture of sp2 and sp3 carbon bonds) [152]. DLC layers have some properties similar to diamond, but they are achieved in an isotropic disordered thin film without grain boundaries [153]. It can also be defined as a composite composed of nanocrystalline diamonds and/or amorphous aluminum with or without hydrogen [152]. Typical properties of DLC layers include extreme hardness, density, electrical resistance, chemical resistance, IR transparency, excellent smoothness, low coefficient of friction, and a high resistance to wear and corrosion [153,154,155]. DLC consists not only of amorphous carbons (a-C) but also of hydrogenated alloys, α-C:H. The hardest form is considered to be quadrilateral amorphous carbon (ta-C). When used already in the form of a two μm thick layer, it increases the service life of stainless steel from 1 week to 85 years [156]. The production of DLC coatings is much cheaper than diamond alone, which is an advantage for many applications. DLC layers and foils are used as protective coatings in biomedicine and electronics, and in the automotive, shipbuilding, and machining industries. DLC layers can be prepared by a variety of methods, such as by physical vapor deposition, magnetron sputtering (PVD) processes, such as sputtering or arc evaporation, or by plasma-enhanced chemical vapor deposition (PECVD) and chemical vapor deposition (CVD) [157,158,159,160,161]. Thus, it is possible to prepare films with specific desired properties located between the properties of graphite and diamond [162]. The resulting properties and quality of the DLC film depend on the substrate, the type of deposition, and the deposition conditions.

Carbon layers can be synthesized in various ways and by different methods, including magnetron sputtering, plasma-enhanced chemical vapor deposition, and ion beam deposition. A straightforward and effective process of carbon layers preparation is evaporation from a graphite source (e.g., carbon filament) [161,163,164]. This method can be used to prepare both very thin layers that are continuous–continuous (thickness value is in the order of tens of nm) and layers of higher thicknesses (in the order of hundreds of nm). The advantage of this method is a very short deposition time, during which there is no contamination of the resulting layers. Important parameters influencing the quality, thickness, homogeneity, and stability of the layers are the rate of deposition and the distance of the substrate from the graphite source. Possible applications of coatings prepared by vapor deposition depend on their parameters and properties, especially on their thickness and mechanical properties. These are crucial and define whether the layers will be useful in electronics or tissue engineering [165,166].

**Figure 9:**
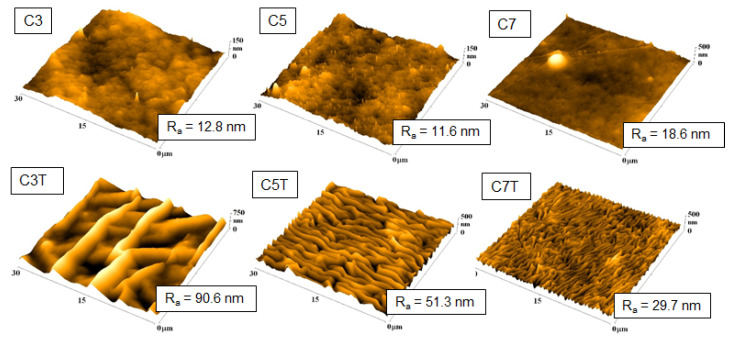
AFM scans of carbon-deposited PLLA before and after thermal treatment. The deposition distances were 3, 5, and 7 cm. The R_a_ value is in nm. The mean roughness value (R_a_) represents the arithmetic average of the deviation from the center plane of the sample Reprinted with the permission from ref. [151], 2019, Elsevier.

Carbon layers are often used in regenerative medicine. However, unmodified carbon often acts as a bioinert, which can be a disadvantage in many biological applications. Therefore, it is appropriate to modify such surfaces further to prepare custom materials—whether surfaces are suitable for cell colonization or, conversely, materials where cell adhesion is undesirable (e.g., surfaces in contact with blood, heart valves, etc.). A number of methods can be used for such treatment—it is possible to use plasma or laser radiation, or thermal lubrication of the prepared layers is often sufficient to nanostructure the surface (Figure 9) [151].

### 2.14. Hybrid Carbon Layers

A very interesting group of materials are hybrid materials. In this case, structures that were created by combining/layering individual types of materials, such as carbon (carbon layers, graphene layers) and noble metal, on top of each other. The resulting materials are not usually used in this original form. Very often, they are subjected to modification by UV or laser radiation. The action of, for example, excimer laser can lead to the interweaving of individual layers and the formation of metal clusters and nanostructures. Structures prepared in this way can be used, for example, in the field of optics, transistors, or sensor applications. The resulting properties of the structure and its “appearance” are strongly dependent on the substrate used and the parameters of the individual layers. It was found that, in the case of a combination of carbon and gold layer with increasing carbon thickness, a wrinkle-type transformation was performed to a homogeneous series in gold nanoclusters [167].

## 3. Conclusions

The properties of carbon nanoparticles, nanolayers and other nanostructures, and their composites in polymeric and other materials have been described in detail in this review. The specific conditions of nanostructures preparation lead to significantly different unique properties of the carbon element. Not only the properties and composites of typical carbon nanostructures, such as carbon nanoparticles, fullerenes, DLC layers, and graphene, have been discussed; new unique carbon forms, such as Q-carbon, have also been introduced. The composites of carbon nanostructures have applications in electronics, sensorics, and tissue engineering. The development of activated graphene, quenched carbon, and other recent discoveries in the field of carbon materials promise that, in this field, new discoveries, with a wide range of future applications, are still possible.
